# Melon (*Cucumis melo*) fruit-specific monoterpene synthase

**DOI:** 10.1186/s43897-023-00051-6

**Published:** 2023-03-03

**Authors:** Kathrine H. Davidson, Syamkumar Sivasankara Pillai, Yukihiro Nagashima, Jashbir Singh, Rita Metrani, Kevin M. Crosby, John Jifon, Bhimanagouda Patil, Seyednami Niyakan, Xiaoning Qian, Hisashi Koiwa

**Affiliations:** 1 Department of Horticultural Sciences, Vegetable and Fruit Improvement Center, Texas A&M University, TX 77843 College Station, USA; 2 Texas A&M AgriLife Research and Extension Center, 2415 E Business 83, Weslaco, TX 78596 USA; 3 Department of Food Science and Technology, Texas A&M University, College Station, TX 77843 USA; 4 Department of Electrical and Computer Engineering, Texas A&M University, College Station, TX 77843 USA; 5 TEES-AgriLife Center for Bioinformatics & Genomic Systems Engineering, Texas A&M University, College Station, TX 77843 USA; 6 Department of Computer Science and Engineering, Texas A&M University, College Station, TX 77843 USA; 7 Molecular and Environmental Plant Sciences, Texas A&M University, College Station, TX 77843 USA

Muskmelon (*Cucumis melo L.*) is one of the important horticultural crops of the Cucurbitaceae family. Global production of melon fruits was approximately 27 million tons, with the United States production yielding 616,050 tons (FAO 2018). Melon is diploid (2n = 24) and has an approximate genome size of 450 Mbp (Arumuganathan and Earle 1991). A high-quality reference genome of melon (DHL92 v4.0) covers 358 Mbp pseudomolecules (Castanera et al. 2019).

Monoterpenes are constituents of plant volatile organic compounds (VOC) and are ten-carbon aromatic compounds. The protective role of terpenoids in foods has been suggested because of the broad antimicrobial activity of terpenoids, which led to the application of terpenoid-containing essential oils for food preservatives. Monoterpenes are formed from the C_5_ precursor, isopentenyl pyrophosphate (IPP), produced through the methylerythritol phosphate pathway (MEP) in the chloroplast and converted to geranyl pyrophosphate (GPP—C_10_) by plastidial GPP synthase. Monoterpene synthases (MTPS) synthesize monoterpenes from GPP. The large family of terpene synthases has been divided into eight clades (TPS-a to TPS-h) (Jiang et al. 2019), in which MTPS typically belongs to TPS-b. We have previously identified 36 terpenoids during melon fruit ripening, of which fifteen were monoterpenes (Nagashima et al. 2021). We also identified MTPS expressed in fruits. However, because MTPS are often promiscuous, and can generate more than one product, the prediction of MTPS responsible for the production of various fruit monoterpenes has been challenging.

To determine genes responsible for various monoterpenes identified in melon fruit, we conducted a bioinformatic survey of MTPS genes in reference strain DHL92 using spearmint (*Mentha spicata*) L-limonene synthase (GenBank accession No. L13459) as a query sequence. The genome annotation of each hit was inspected and corrected using RNA-seq data, resulting MTPS1-MTPS9, which are clustered on chromosome 11 (Fig. 1A, Supplemental Data1). The sequence relationship of MTPS was visualized by protein sequence alignment and phylogenetic tree analysis (Fig. 1B, Supplemental Fig. 1). As a reference, Spearmint and Citrus limonene synthases, which produce L- and D-limonene, respectively, were included in the analysis. Melon MTPS forms four groups (1–4) based on the amino acid sequence similarity. In the phylogenetic tree, group 1 MTPS was the closest, whereas group 4 is the most distant from the known LS proteins.

**Fig. 1:**
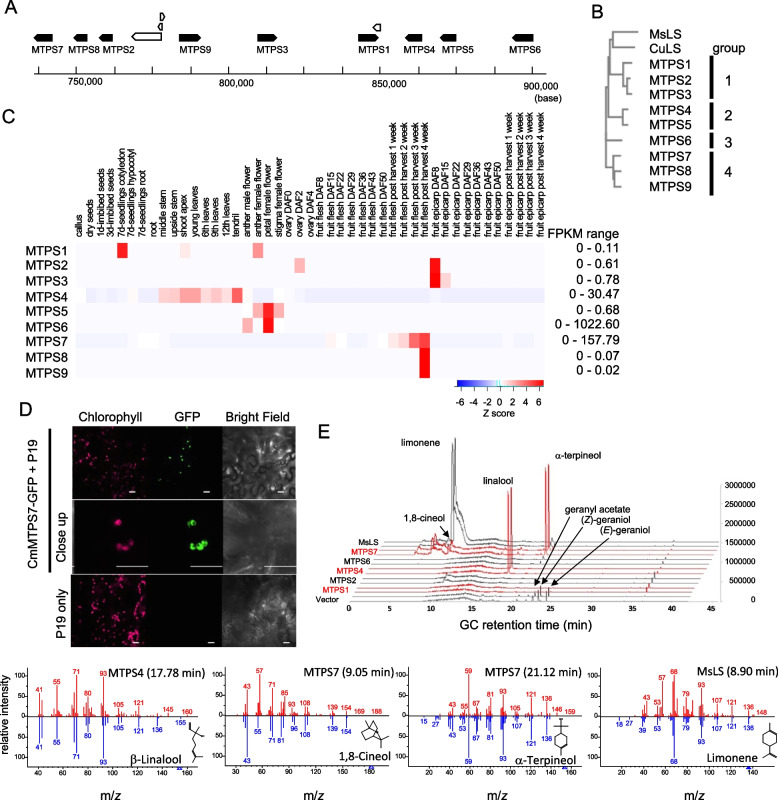
**A** Genomic organization of the MTPS cluster on chromosome 11. Filled box arrows indicate the positions of MTPS coding sequences. **B** Phylogenetic tree of MTPS. MTPS1, Melo3C023284; MTPS2, Melo3C023278; MTPS3, Melo3C023282; MTPS4, Melo3C023286; MTPS5, MELO3C023287; MTPS6, Melo3C023288; MTPS7, Melo3C023275; MTPS8, Melo3C023276; MTPS9, Melo3C034557; MsLS Mentha spica L-limonene synthase (GenBank accession number L13459); CuLS, Citrus unshiu d-limonene synthase (GenBank accession number BAD27257). **C** Heat map showing expression profile of MTPSs using RNA seq data (Yano et al. 2020). **D** Subcellular localization of MTPS7-GFP. Leaves of *N. benthamiana* were co-infused with Agrobacterium tumefaciens cells containing MTPS7-GFP plasmid and cells containing the 35S-P19 plasmid. Control plants were infused only with cells containing 35S-P19. Images were obtained three days after infiltration. **E** Selected-ion chromatogram (*m/z* = 92.5–93.5) of terpenoids produced by *E. coli* cells expressing MTPS isoforms. Each cell line was cultured in duplicates in the presence of 1/10 culture volume of dodecane. Terpenoids extracted in dodecane fraction were separated by GC–MS. Peaks corresponding to major terpenoids were labeled. The bottom panels show MS spectra for the identified peaks (blue) with the spectra of standards (red) in National Institute of Standards Mass Spectral Library

The expression of genes in a gene cluster is often non-uniform. We have previously shown that MTPS7 (Melo3C023275) is highly expressed during fruit development of Western shipper (F39) and Tuscan (da Vinci) varieties (Nagashima et al. 2021).  Expression of other MTPS was relatively low in fruits. To gain a broader gene expression profile of melon MTPS, we generated a digital expression profile using RNA-seq data (Fig. 1C). The expression profile largely follows the phylogenetic relationship. In group 1, MTPS1 expresses mainly in cotyledon and shoot meristem, and MTPS2/MTPS3 express in epicarp (fruit skin). Group 2 (MTPS4/MTPS5) expresses in leaf and stem, but MTPS5 also expresses in flowers like group 3 (MTPS6). Group 4 genes (MTPS7/MTPS8/MTPS9) express in fruits; however, MTPS7 predominates the expression levels. Overall, this indicated that MTPS7 is a predominant MTPS in fruits. Based on FPKM values, MTPS7 expression reaches 162, whereas other MTPSs only express at 0.02–1.6. These results confirm that MTPS7 is a predominant fruit MTPS across different varieties.

The fruit-specific isoform, MTPS7, was functionally characterized. Plastid localization of MTPS7 was confirmed using transient expression of MTPS7-GFP in *Nicotiana benthamiana* (Fig. 1D). GFP-MTPS7 accumulated oval-shaped structures that overlap with chlorophyll autofluorescence. To understand the product specificity of MTPS7, we bacterially expressed MTPS7 for metabolite analysis (Alonso-Gutierrez et al. 2013). Additionally, we included representative isoforms of other MTPS groups, namely, MTPS1, MTPS2, MTPS4, and MTPS6 in the analysis. The truncated cDNAs corresponding to the sequences in Supplemental Fig. 1 were cloned into pTrc-trGPPS(CO)-LS replacing the limonene synthase ORF (identical to the MsLS sequence in Supplemental Fig. 1) in the vector. We chose to use the partial truncation of transit peptide for each MTPS because the corresponding truncated MsLS was successfully expressed in this system in the previous study (Alonso-Gutierrez et al. 2013). When co-transformed into host cells (DH10B) with JBEI3085, geranyl diphosphate is supplied from the host cells. Production of monoterpenes in the transformed cells was measured by GC–MS. Selected ion chromatograms for m/z = 93 corresponding terpenoid fragments are shown in Fig. 1E. A negative control that expresses the MVA pathway components and GPPS but not any terpene synthase transgene produced a small amount of geraniol isoforms (retention time 23.4 min and 24.4 min) and geranyl acetate (retention time 22.7 min), perhaps due to endogenous phosphatase (Liu et al. 2015) and promiscuous activity of chloramphenicol acetyltransferase (Alonso-Gutierrez et al. 2013). Positive control produced limonene (Alonso-Gutierrez et al. 2013) (retention time 8.90 min). Of the five MTPS tested, MTPS4 produced linalool (retention time 17.78 min), and MTPS7, the MTPS in fruit, predominantly produced 1,8-cineol and α-αterpineol (retention time 9.05 min and 21.12 min). This is consistent with the high level of α-terpineol detected throughout the fruit development (Nagashima et al. 2021). On the other hand, using this system, we could not detect specific monoterpene production in seedling-specific MTPS1, epicarp-specific MTPS2, and flower-specific MTPS6. However, we could not conclude if the apparent lack of activities was due to technical causes associated with the bacterial expression system used. Additional monoterpenoids, citronellol, citronellal, citral, and geraniol were detected in some recombinant cultures; however, these compounds were found in very small amounts and also in the negative control strain, likely by the action of endogenous phosphatases (Wang et al. 2016); therefore, it was not possible to determine if they are produced by MTPS proteins expressed. A recent study of terpene synthases in cucumber (*Cucumis sativas*) (He et al. 2022) identified eleven MTPS-like genes, eleven sesquiterpene synthase-like (STPS-like), and two diterpene synthase-like genes in the cucumber genome sequence. Interestingly, several MTPS and STPS-like proteins in cucumber could use both GPP and FPP as substrates and produce both monoterpenes and sesquiterpenes. In the melon genome, fourteen STPS-like genes are present, which may contribute to additional mono and sesquiterpene productions.

Volatile terpenoids are essential oil ingredients used for bactericidal, virucidal, fungicidal, antiparasitic, insecticidal, medicinal, and antioxidant applications. Consistently, some cucumber TPS are induced by herbivore attacks (He et al. 2022). Given such properties, it is reasonable to assume fruit-specific monoterpenes are acting as natural preservatives to protect fruits from microbial damage. Our previous data showed that α-terpineol was a major fruit monoterpene that persisted throughout the fruit development. Relatively high levels of α-terpineol were detected at the early stages of fruit development, where MTPS7 expression was relatively low. This may indicate contributions of other isoforms or sufficiency of MTPS7 expression levels due to high α-terpineol production by this isoform. In cucumber, α-terpineol was produced by CsTPS10, CsTPS11, and CsTPS15 (He et al. 2022); however, in vitro CsTPS activity showed high promiscuity, contrasting with our observation that each MTPS formed only 1 or 2 monoterpenes. This may be due to different assay systems (in vivo production in *E. coli* vs. in vitro assay using purified *E. coli-*produced enzyme, and may indicate that product specificity of monoterpene synthase is higher in vivo than in vitro.

In conclusion, we established that MTPS7 is a fruit-specific isoform and produces predominant fruit monoterpenoids. These findings are milestones to improving melons with aromatic flavors and antibacterial activities associated with these monoterpenoids for better qualities and food safety (Guimarães et al. 2019).

### Supplementary Information

**Additional file 1:** **Supplemental Figure 1.** Alignment of melon MTPS. Arrowhead indicates predicted cleavage site for transit peptide. Transit peptide regions with low homologies (corresponding to amino acids 1-57 of MsLS) were excluded from the alignment. Arrowhead indicates cleavage site for transit peptide for MsLS. The red box indicates a conserved catalytic motif (DDxxD).

## Data Availability

The datasets used during the current study are available from the corresponding author upon reasonable request.
