# Supplementary material for: Melon (Cucumis melo) fruit-specific monoterpene synthase
Source: Mol Hortic. 2023 Mar 3;3:3. doi: 10.1186/s43897-023-00051-6 (PMC10514926; doi:10.1186/s43897-023-00051-6)
Supplement: Supplementary file 1 — Additional file 1: Supplemental Figure 1. Alignment of melon MTPS. Arrowhead indicates predicted cleavage site for transit peptide. Transit peptide regions with low homologies (corresponding to amino acids 1-57 of MsLS) were excluded from the alignment. Arrowhead indicates cleavage site for transit peptide for MsLS. The red box indicates a conserved catalytic motif (DDxxD). [file 43897_2023_51_MOESM1_ESM.pdf]

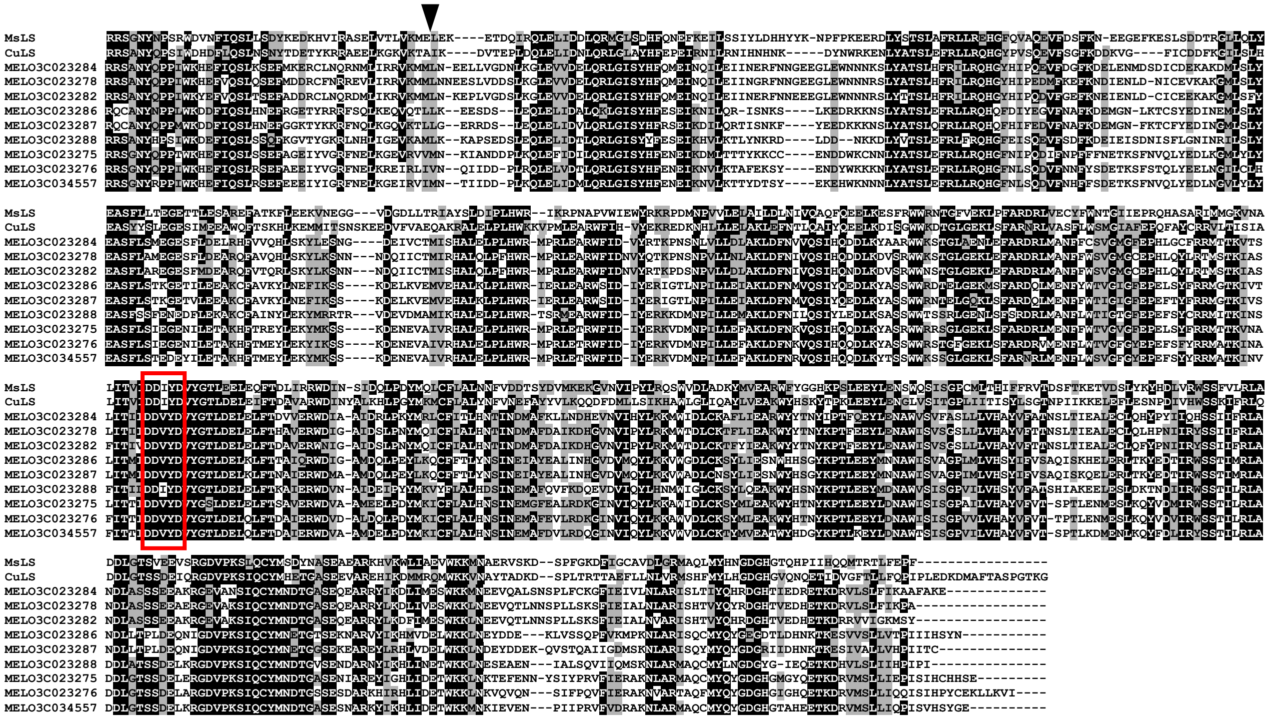

Supplemental Figure 1. Alignment of melon MTPS. Arrowhead indicates predicted cleavage site for transit peptide. Transit peptide regions with low homologies (corresponding to amino acids 1-57 of MsLS) were excluded from the alignment. Arrowhead indicates cleavage site for transit peptide for MsLS. The red box indicates a conserved catalytic motif (DDxxD).

Supplement

Methods

Construction of plant and bacterial expression clones

Coding sequences of MTPS were synthesized by Twist bioscience (USA) and subsequently amplified by PCR with flanking BamHI and SalI sites. The PCR products were cloned into pTrc-trGPPS(CO)-LS (Addgene #50603) between BamHI and SalI, replacing *Mentha spicata* limonene synthase, to produce pTrcMTPS. To prepare GFP-MTPS, PCR products of MTPS and linker-GFP were inserted into pEnEOimCherryF3SGThsp (GenBank accession No. KF537341) between NcoI-NotI sites. Subsequently, pEnEOiMTPS-linkGFPThsp was recombined with pMDC99 (Curtis and Grossniklaus 2003) using Gateway LR clonase II (Thermo Fisher) according to the manufacturer's protocol to produce pMDC99MTPS-GFP. pMDC99MTPS-GFP was introduced into *Agrobacterium tumefaciens* GV3101 and was used for plant transient expression.

Agroinfiltration and microscopy

Nicotiana benthamiana host plants were grown in potting mix (Jolly Gardener C/20) in 4" pots for four weeks under 23°C, 16h light /8h dark conditions. Infiltration of *Agrobacterium tumefaciens* cultures with pMDC99MTPS-GFP were performed as described previously (Fukudome et al. 2014).

Bacterial production of monoterpenoids and GC-MS analysis

pTrcMTPS and JBEI-3085 (Addgene No.87950) were co-transformed into DH10B. Transformed cells were inoculated to LB medium containing 100 mg/L ampicillin and 25 mg/L chloramphenicol at 37°C for overnight to prepare preculture. Induction cultures were prepared by inoculating 0.5 ml overnight preculture to 50 ml LB medium containing 100 mg/L ampicillin and 25 mg/L chloramphenicol, and 5 ml dodecane. Production cultures were shaken at 37°C until OD600=0.6. The cultures were then cooled to 30°C, and the final 0.1 mM IPTG was added. The cultures were shaken at 30°C for 20-24 hours before harvest.

For GC-MS analysis, the harvested cultures were centrifuged, and the dodecane fraction was recovered. Volatile analysis was carried out as described previously (Metrani et al. 2022). Briefly, samples were analyzed by TRACE GC Ultra coupled with TriPlus Autosampler, DSQ II Mass Spectrometer (Thermo Fisher Scientifc, Inc., USA), and Restek Rtx-Wax column (30 m × 0.25 mm ID with 0.25 µm film thickness; Restek Corp., USA). Samples were kept at a

thermostatic box at 60 °C under constant agitation for 30 min using an autosampler and volatile were extracted using 50/30 μm divinylbenzene/carboxen/ polydimethylsiloxane fiber. The injector port was kept at 225°C and helium was used as a carrier gas (flow rate 1ml/min). The initial oven temperature was held at 40 °C for 2 min, then increased to 210 °C at a rate of 5 °C/min, and then held for 6 min. The mass detector was operated at 70 eV with the electron ionization mode source kept at 285 °C. Spectra were recorded (mass range of m/z 30–300) with a scan rate of 15.6 scans/sec. Data were processed with Thermo Xcalibur software (Thermo Fisher Scientific, USA). Nootketone was used as an internal standard. Identification of volatile compounds was achieved by matching the mass spectra database National Institute of Standards and Technology (NIST MS search 2.0) and n-alkanes (C5–C24).

References

Curtis MD and Grossniklaus U (2003). A gateway cloning vector set for high-throughput functional analysis of genes in planta. Plant Physiol 133: 462-469. 10.1104/pp.103.027979

Fukudome A, Aksoy E, Wu X, Kumar K, Jeong IS, May K, Russell WK and Koiwa H (2014). Arabidopsis CPL4 is an essential C-terminal domain phosphatase that suppresses xenobiotic stress responses. Plant J 80: 27-39. 10.1111/tpj.12612

Metrani R, Jayaprakasha GK and Patil BS (2022). Optimization of Experimental Parameters and Chemometrics Approach to Identify Potential Volatile Markers in Seven Cucumis melo Varieties Using HS–SPME–GC–MS. Food Analytical Methods 15: 607-624. 10.1007/s12161-021-02119-9

Supplemental Data1. MTPS sequence FASTA file

>MTPS1 [organism=Cucumis melo] Melo3C023284 monoterpene synthase 1
ATGAGTATTCTCCATCTTCTCCTTCCTCTTTATCCTTCCATCTCTTGTTTTACCTCCACACAAAATCTTCCCTC
TTCTCCAAACTTTTATGTTCTTTGCCAAATGGATAAGCCATCCATGGTCATCAAAAAACTTTCTCATAATTC
TGTTGTTCGACGATCTGCAAACTACCAACCTCCTATTTGGAAACATGAGTTTATTCAATCCTTGAAGAGTG
AATTCATGAAGGAGAGGTGTTTAAATCAAAGGAATATGCTAATAAGAAGAGTGAAAATGATGCTCAAT
GAAGAATTATTGGTTGGTGATAATTTGAAAGGATTGGAGGTTGTTGATGAATTGCAAAGGTTGGGAATC
TCATATCACTTTCAAATGGAAATAAATCAAATATTAGAGATTATCAATGAAAGATTCAACAATGGAGAAG
AAGGTTTGGAATGGAACAACAACAAAAGCTTATATGCCACATCTCTTCATTTTAGAATTTTAAGGCAACA
TGGCTATCACATCCCTCAAGAGGTATTTGACGGGTTTAAGGATGAACTAGAAAATATGGATAGTGATAT
TTGTGATGAGAAAGCTAAAGATATGTTATCATTGTACGAAGCTTCCTTCTTGTCAATGGAAGGTGAAAGC
TTTTTGGATGAACTGAGGCACTTTGTCGTTCAACATCTTTCAAAATACCTTGAATCAAATGGTGATGAAAT
AGTTTGTACTATGATAAGCCACGCTCTTGAACTCCCACTTCATTGGAGAATGCCAAGATTGGAGGCTAGA
TGGTTTATCGATGTGTACCGAACAAAACCCAACTCAAATCTAGTGTTGTTGGATTTAGCCAAGTTAGATT
TCAACGTTGTCCAATCCATACATCAAGATGACCTCAAATATGCTGCTAGGTGGTGGAAGAGTACAGGGC
TTGCAGAAAACCTAGAGTTTGCAAGGGATAGACTAATGGCTAATTTCTTTTGCTCTGTGGGAATGGGATT
TGAACCTCATCTTGGATGTTTTAGAAGAATGACCACAAAAGTTACTTCATTAATAACAATAATTGATGAT
GTTTATGATGTCTATGGAACTTTGGACGAGCTCGAACTCTTTACCGATGTCGTTGAGAGATGGGATATTG
CAGCAATTGATCGCTTGCCTAAATACATGAGATTATGTTTTATTACTCTCCACAACACCATCAACGATATG
GCTTTCAAACTACTCAATGATCATGAAGTCAATGTCATTCATTATTTAAAAAAAATGTGGATAGATTTATG
CAAAGCATTTTTGATAGAAGCAAGGTGGTATTACACAAATTACATACCAACATTTCAAGAGTATTTAGAG
AATGCTTGGGTTTCGGTATTTGCATCTCTTCTTCTTGTTCATGCTTATGTCTTTGCCACAAACTCATTGACA
ATTGAGGCTTTGGAATGCTTGCAACATTATCCTTATATTATTCAACATTCATCCATCATATTTCGTCTTGCC
AATGATTTAGCTTCATCATCGGAGGAAGCAAAGAGAGGAGAGGTCGCTAATTCTATACAATGTTACATG
AATGACACGGGTGCATCAGAACAAGAAGCAAGACGATACATAAAGGATTTGATTATGGAATCGTGGAA
AAAAATGAATGAAGAAGTTCAAGCTTTGAGTAATTCACCATTATTCTGTAAGgGTTTCATTGAAATTGTTT
TAAATTTGGCTAGAATTTCTCTTACTATCTACCAACATAGAGATGGGCACACTATCGAAGATCGTGAGAC
TAAGGATCGTGTATTATCTTTATTTATCAAGGCCGCTTTTGCAAAAGAGTGA
>MTPS2 [organism=Cucumis melo] Melo3C023278 monoterpene synthase 2
ATGACAATGGCTATTCTTCATCATCCTCTTCTTTCCCCCATCTCTTTTTTCACCTCCACACAAAACAGTCTTC
CTTCTCATCCTAAATTCTATGTTTTTTCTCAAATCGTTAAACAATCGCCTACGGTTCGAAAAGCTTGCAATG
ATTCTGTCGTCCGACGATCTGCAAATTACCAACCTCCTATTTGGAAGCATGAGTTTGTTCAATCCTTGCAG
AGTGAATTCATGGATGATAGATGTTTTAATCGAAGGGAGGTGCTAATAAGAAGAGTGAAAATGATGCT
CAATAATGAAGAATCATTGGTTGATGATAGTTTGAAAGGATTGGAAGTTGTTGATGAATTGCAAAGATT
GGGAATCTCATATCACTTTCAAATGGAAATAAATCAAATATTAGAGATTATCAATGGAAGATTCAACAAT
GGAGAAGAAGGTTTAGAATGGAACAACAACAGAAGTTTATATGCCACATCTCTCCATTTTAGAATTTTAA
GGCAACATGGCTATCACATCCCTGAAGACATGTTCAAGGAGTTTAAGAATGACATAGAAAATTTGGATA
ATATTTGTGAGGTGAAAGCTAAAGGAATGTTATCATTGTATGAAGCTTCATTCTTAGCAATGGAAGGTG
AAAGCTTTTTGGATGAAGCCAGACAATTTGCCGTCCAACATCTTTCAAAATACCTTAAATCTAATAATAAT
GATCAAATCATTTGTACTATGATAAGACATGCTCTTCAACTCCCATTTCATTGGAGAATGCCAAGATTGG
AGGCTAGGTGGTTTATCGATAATGTGTATCAAACAAAACCCAACTCAAATCCGGTGTTGTTGAATTTAGC
CAAATTAGACTTCAACATTGTCCAATCCATTCATCAAGATGACCTAAAAGATGTCTCTAGGTGGTGGAAG
AGCACAGGGCTTGGAGAGAAACTTGAGTTTGCAAGAGATAGATTGATGGCTAATTTCTTTTGGTCTGTG

GGAATGGGATGTGAACCTCATCTTCAATATCTTAGAACAATGTCTACAAAAATTGCTTCATTAATAACCA
TTATTGATGATGTTTATGACGTCTATGGAACTCTGGACGAACTTGAACTTTTTACCCATGCTGTTGAGAG
ATGGGATATCGGAGCAATTGATTCATTACCGAACTACATGCAAATATGTTTTATTGCTCTCCACAACACC
ATCAATGACATGGCTTTTGATGCAATAAAGGATCATGGAGTGAATGTCATTCCATATTTAAGAAAAATGT
GGACAGATTTATGCAAAACATTCTTGATCGAAGCAAAGTGGTATTACACAAATTACAAACCAACATTTGA
AGAGTACTTAGAGAATGCATGGATTTCAGTATCAGGATCTCTTCTTCTTGTTCATGCTTATGTCTTCACCA
CAAATTCATTAACAATAGAGGCTTTGGAGTGCTTGCAACTTCATCCAAATATCATTCGATATTCATCCATC
ATCTTTCGTCTTGCCAATGATTTAGCTTCATCTTCGGAAGAAGCAGAGAGAGGAGAAGTCGCTAAATCTA
TACAATGTTACATGAATGACACGGGTGCTTCAGAACAAGAAGCACGACGATATCTTAAGGATTTGATTG
TGGAATCATGGAAGAAATTGAATGAAGAAGTTCAAACTTTGAATAATTCACCATTACTCTCCAAAAGTTT
CATTGAAATTGCGTTAAATCTTGCTAGAATTTCTCATACTGTCTACCAATATAGAGATGGGCACACTGTTG
AAGATCATGAGACCAAGGATCGTGTATTATCCTTGTTTATCAAGCCTGCTtaa
>MTPS3 [organism=Cucumis melo] Melo3C023282 monoterpene synthase 3
ATGAAAACGAGTCTTCTTCAGCTTTCTCTTCTTTCCCCCATTTCTTGCTTCGCCTCCACACAAAACAATATT
CCTAGTTCTTATCCTAAGTTTTATGTTTTTTCCCAAATGGCTAAGTCGTCGGCTAATTATGTTCGAAACGG
TTACGACGACTTTGTCGTTCGACGATGTGCAAATTACCAACCTCCTATTTGGAAGTATGAATTTGTTCAAT
CCTTGACGAGTGAATTCATGGACGACAGATGTTTAATTCGAAGGGATATGCTAATAAAAAGAGTGAAAA
TGATGCTCAACAAAGAACCATTGGTTGGTGATAGTTTGAAAGGATTGGAAGTTATTGATGAATTGCAAA
GGTTGGGAATCTCTTATCACTTTCAAATGGAAATAAATCAAATGTTAGAGATTATCAATGAAAGATTCAA
CAATGGAGAAGAAGGTTTAGAATGGAATAACAACAGAAGTTTATATGCCACATCTCTTCATTTTAGAATT
TTAAGGCAACATGGCTATCACATCCCTCAAGACGTGTTCAAGGAGTTTAAGAATGAGATAGAAAATTTG
GATTGTATTTGTGAAGAGAAAGCTAAAGGAATGTTAACATTTTATGAAGCTTCCTTCTTAGCAATGAAAG
GCGAAAGCTTTGTGGATGAAGCCAGACAATTTGCCATCCAACGTCTTTCAAAATATCTTAAATCTAATAA
TAATGATCAAATAATTTGTACTATGATAAGCCATGCTCTTCAACTCCCATTTCATTGGAGAATGCCAAGAT
TGGAGGCTAGGTGGTTCATCGATAACGTGTATCGAACAAAGCCCAACTCAAATCCGGTGTTATTGGATT
TAGCCAAATTAGACTTCAACATTGTCCAATCTATTCATCAAAATGATCTAAAAGATGTCTCCAGGTGGTG
GAAGAGCACAGGGCTTGGAGAGAAACTTGAGTTTGCAAGAGATAGATTGATGGCTAATTTCTTTTGGTC
TGTGGGAATGGGATGTGAACCTCATCTTCAATATCTTAGAACAATGTCTACAAAAACTGCTTCATTAATA
ACCATTATTGATGATGTTTACGACGTCTATGGAACTTTGGACGAACTTGAACTTTTTACCGATGCTGTTGA
GAGATGGAATATCGGTGCAATTGATTCATTACCGAACTACATGCAAATATGTTTTATTGCTCTCCACAAC
ACCATCAATGACATGGCTTTTGATGCAATAAAGAATCATGGAGTCAATGTCATTCCATATTTAAGAAAAA
CGTGGACAGATTTATGCAAAACATTCTTGATCGAAGCAAAGTGGTATTACACAAATTACAAACCAACATT
TGAAGAGTACTTAGAGAATGCATGGATTTCAGTATCAGGATCTCTTCTTCTTGTTCATGCTTACGTCTTCA
CCACAAATTCATTAACAATAGAGGCTTTGGAGTGCTTGCAACTTTATCCAAATATTATTCGATATTCATCC
ATTATCTTTCGTCTTGCCAATGACTTAGCTTCATCTTCGGAAGAAGCAAAGAGAGGAGAAGTCGCTAAAT
CTATACAATGTTACATGAATGACACGGGTGCTTCAGAACAAGAAGCACGACGATATATTAAGGATTTAA
TTATGGAATCATGGAAGAAATTGAATGAAGAAGTTCAAACTTTGAATAATTCACCATTACTCTTAAAAGA
TTTCATTGAAATTGCGTTAAATCTTGCTAGAATTTCTCATACTGTCTACCAACATAGAGATGGTCACACTG
TTGAAGATCATGAGACCAAGGATCGTGTATTATCTTTATTTATGAAGTCTGCTTAG
>MTPS4 [organism=Cucumis melo] Melo3C023286 monoterpene synthase 4
ATGCTCTCAATGGCTCTTCTCCACTATCCTCTTTCTTCTGCATTCTCTTTTCATGCAGCTGCTCTACCATCTA
CAAACTATCAACCTTCTTTTACTATGTTAAAATATGTTGTGGTTGAACGTGGAATGTGCATAAAAGCATC
GATACAAACTCAAAGTGGTGATGTTATTGTGAGGCAATGTGCAAATTACAATCCTCCTTTGTGGAAAGAT
GATTTTATTCAATCATTACATAATGAATTCAGGGGAGAAACATATAGAAGACGATTTAGTCAATTAAAAG

AACAAGTTCAAACATTGCTGAAAGAAGAAAGTGACTCTTTGGAGCAGCTCGAACTCATTGATGCCTTAC
AAAAGCTTGGAATATCATACCACTTTGAGAGTGAAATTAAAAATATACTACAAAGAATAAGCAACAAGT
CTCTTAAAGAGGATAGGAAGAAGAATAGTCTCTACGCAACATCTCTTGAATTTCGACTTCTAAGACAACA
CCAATTTGATATTTATGAAGGTGTTTTTAATGCCTTCAAAGATGAGATGGGGAATTTAAAAACATGCTCT
TATGAAGATATAAATGAAATGTTATCTTTATATGAAGCTTCATTCTTATCAACTAAAGGGGAGACTATTTT
GGAGGAAGCAAAATGCTTTGCAGTAAAATATCTAAATGAATTCATCAAATCAAGCAAAGATGAACTCAA
AGTAGAGATGGTAGAGCATGCCTTGAAGCTTCCTTTACACTGGAGAATAGAAAGATTGGAGGCAAGAT
GGAGTATTGATATATATGAGAGAATAGGAACCCTAAATCCTATTCTTCTTGAAATTGCTAAACTTGATTTC
AACATGGTGCAATCTATTTACCAAGAAGACCTTAAGTATGCATCAAGTTGGTGGAGAGACACAGAGCTA
GGAGAAAAGATGAGCTTTGCAAGAGACCAACTGATGGAAAATTTCTATTGGACAGTAGGCATTGGATTT
GAACCTGAGCTTTCATATTTTAGAAGAATGGGCACAAAGATTGTGACATTGATTACAATGATTGATGATG
TTTATGATGTCTATGGCACATTGGATGAACTCAAACTCTTTACAACTGCAATACAGAGGTGGGATATTGG
AGCAATGGACCAACTTCCTGAGTACTTGAAACAATGTTTTTTTACTCTCTACAATTCAATAAATGAGATAG
CTTATGAAGCACTAATCAATCATGGAGTTGATGTCATGCAATACCTCAAGAAAGTGTGGGGAGATTTGT
GCAAATCTTATTTAATAGAGTCAAATTGGCATCACAGTGGCTATAAACCAACGTTGGAAGAATATATGAA
CAATGCATGGATATCAGTAGCAGGACCACTTATGTTAGTTCATTCATATATTTTTGTATCAGCTCAAATAT
CAAAGCATGAATTGGAGAGATTGACAAAATATGAAGATACAATTCGTTGGTCATCAACAATTATGCGAC
TTGCAAATGACCTATTAACACCATTGGATGAACAAAACATTGGTGATGTTCCAAAATCAATTCAATGCTA
CATGAATGAAACAGGCACTTCAGAGAAAAATGCTCGTGTTTACATAAAACATATGGTAGATGAGTTATG
GAAGAAGTTAAATGAATATGATGATGAGAAACTTGTGTCCTCACAACCCTTCGTCAAGATGCCTAAGAA
TCTGGCACGAATATCTCAATGTATGTATCAATATGGAGAAGGAGATACATTAGATCACAACAAGACAAA
AGAAAGTGTGGTGTCTCTTCTTGTAACACCTATTATCATTCATTCATATAACTAA
>MTPS5 [organism=Cucumis melo] Melo3C023287 monoterpene synthase 5
ATGTCTCTCATTCGCTTCCATCTTCCCATTTCTAAATTGTCCGTCTCCAATCTTGTTTCTAACGATTGCAAC
GTTCGACGATATGCAAACTATCAATCTCTTTTTGTAATGTTGAAATCTAATGGAGTTGGAGGTGGGATGT
GCTTAAAAGCTTCAACACAATCTGATGATAATGTTATTGTGAGGCAGTGTGCAAATTATCAACCTCCTAT
GTGGAAAGATGATTTTATTCAATCATTACATAATGAATTTGGGGGAAAAACATATAAAAAACGGTTCAA
CCAACTCAAAGGACAAGTTAAGACATTGCTTGGAGAAAGAAGAGACTCTTTAGAACAACTCGAGCTCAT
TGATGTTTTACAAAGACTTGGAATATCTTACCACTTTAGGAGTGAAATTAAAGATATCTTACAAAGAACT
ATAAGCAACAAGTTCTATGAAGAGGACAAGAAGAAGAATAGTCTTTATGCAACGTCTCTTCAATTTCGAC
TTCTAAGACAACACCAATTTCATATTTTCGAAGATGTTTTCAATGCCTTCAAAGATGAGATGGGGAATTTC
AAAACATGCTTTTATGAAGATATAAATGGAATGTTATCTTTATATGAAGCTTCATTCTTATCAACTAAAGG
GGAGACTGTTTTAGAGGAAGCAAAATGCTTTGCAGTAAAATATCTAAATGAATTCATCAAATCAAGCAA
AGATGAACTCAAAGTAGAGATGGTAGAGCATGCCTTGAAGCTTCCTTTACACTGGAGAATAGAAAGATT
GGAGGCAAGATGGAGTATTGATATATATGAGAGAATAGGAACCCTAAATCCTATTCTTCTTGAAATTGC
TAAACTTGATTTCAACATGGTGCAATCTATTTACCAAGAAGACCTTAAGTATGCATCAAGTTGGTGGAGA
AACACAGAGCTTGGACAAAAATTGAGCTTTGCAAGAGACCAGCTGATGGAAAATTTCTTTTGGACAATT
GGGATTGGTTTTGAACCCGAGTTTACATATTTCAGAAGAATGGGTACAAAGATTGTGTCATTGATAACA
ATGATTGATGATGTTTATGATGTTTATGGCACATTGGATGAACTCAAACTCTTTACAAACGCTATAGAGA
GGTGGGATATGGCAGCAATGGACCAACTTCCTGAGTACTTGAAACAATGTTTTTTTACTCTCTACAATTC
AATAAATGAGATAGCTTATGAAGCACTAATCAACCATGGAGTTGATGTCATGCAATACCTTAAGAAAGT
GTGGGCAGATTTGTGCAACTCTTATTTAATAGAGTCAAATTGGTATCACAGTGGGTATAAACCAACATTG
GAAGAATATATGAACAATGCATGGATATCAGTAGCAGGACCAATTATGTTAGTTCATTCATATATTTTTG
TATCAGCTCAAATATCAAAGCAAGAATTGGAGAGATTGACAAAATATGAAGATACAATTCGTTGGTCAT

CGACAATTATGCGACTTGCAAATGACCTATTAACACCACTGGATGAACAAAACATTGGTGATGTTCCAAA
ATCAATTCAATGCTACATGAATGAGACAGGTGGTTCAGAAAAAGAAGCTCGTGAATATCTAAGACATTT
GGTAGATGAGTTATGGAAGAAGTTAAATGATGAATATGATGATGAGAAACAGGTATCCACTCAAGCCAT
CATTGGCGACATGTCCAAGAATCTGGCACGAATATCTCAATATATGTATCAGTATGGAGATGGGCGAAT
AATCGACCATAACAAAACAAAAGAAAGTATAGTGGCTCTTCTTGTTCATCCTATTATTACTTGTTAA
>MTPS6 [organism=Cucumis melo] Melo3C023288 monoterpene synthase 6
ATGGCTCTTCTCCACCTTCCTCTTCCTTCTACCTTCAAGTTTCATTCTCCTTTGGCCAATGGAGCTCGTCGA
CCATCTTGGAACTATCGACCTTCTTCTATAAGGATCCCTTCCTACTTCATGATCTCTGTTGGGTTTGAATGT
AAGAAGAGAATAAAAGGGCAGTATGAAAGTGAAAATGCCACTGTGAGACGATCTGCAAATTATCATCC
TTCTATTTGGAAAGATGAGTTTATTCAATCCTTAAGCAGTCAATTCAAGGGAGTAACATATGGCAAACGG
TTAAATCACTTGATTGGAGAAGTTAAAGCCATGCTTAAAAAAGCACCAAGTGAAGATTCATTAGAGCAA
TTGGAACTCATTGACACCTTACAAAGACTTGGAATATCATACTACTTTGAGAGTGAAATTAAACATGTAC
TCAAAACATTATACAACAAAAGAGATCTTGATGACAATAAAAAAGATCTTTATGTCACTTCTCTTGAATTC
AGACTATTTAGACAACATGGGTTTGAGATTTCTCAAGAAGTTTTCAGTGACTTCAAGGATGAGATAGAA
ATTTCCGATAATATATCCTTTTTGGGGAATATAAATAGAATTTTATCTTTATATGAAGCATCATTCTCATCG
TTTGAAAATGAAGACTTTTTAGAAAAAGCAAAGTGTTTTGCTATCAATTATTTAGAGAAGTATATGAGAA
GAACAAGGGTGGATGAAGTTGATATGGCCATGATAAAGCATGCTTTGGAACTTCCATTGCATTGGAGGA
CATCAAGAATGGAGGCAAGATGGTTCATTGATATATATGAAAGAAAAAAGGATATGAATCCTATTCTTC
TTGAAATGGCGAAATTAGATTTCAACATTTTACAATCTATCTACCTAGAAGATCTTAAGTCTGCATCAAGT
TGGTGGACGAGCAGTCGACTTGGGGAAAATTTGAGCTTTTCAAGAGACAGATTAATGGCAAATTTTTTA
TGGACAATAGGTACTGGATTTGAACCCGAGTTCTCATATTGTAGAAGAATGATCACAAAGATCAATTCAT
TCATCACAATATTAGATGATATTTATGACGTTTATGGTACATTGGATGAACTTGAGCTCTTTACAAAAGCT
ATAGAGAGATGGGATGTTAATGCAATTGATGAGATTCCTTATTATATGAAAGTGTATTTTCTTGCTCTTCA
TGATTCTATCAATGAGATGGCTTTTCAAGTATTCAAGGACCAAGAAGTTGATGTCATCCAATACCTTCAT
AATATGTGGATAGGTTTGTGCAAATCCTATTTGCAAGAAGCAAAGTGGTATCATAGCAATTACAAACCA
ACACTTGAAGAATATATGGACAATGCATGGGTTTCCATATCCGGACCTGTCATTTTAGTCCATTCATATGT
CTTTGCAACATCTCATATAGCAAAGGAGGAGTTGGAGAGCTTGGACAAAACCAATGACATAATTCGTTG
GTCCTCCACAATTTTACGACTTGCAGACGACCTAGCAACATCATCGGACGAGTTAAAAAGAGGCGACGT
CCCAAAATCAATTCAATGTTACATGAACGACACTGGAGTTTCAGAGAATGATGCTCGTAATTACATAAAA
CATTTGATCAATGAGACATGGAAGAAGCTAAATGAATCCGAAGCTGAAAATATTGCTTTGTCTCAAGTTA
TCATTCAAATGTCTAAGAATCTTGCTCGAATGGCTCAATGCATGTACTTAAATGGAGATGGATACGGTAT
AGAACAAGAAACAAAAGATCATGTATTGTCTTTAATTATTCACCCTATTCCAATTTAA
>MTPS7 [organism=Cucumis melo] Melo3C023275 monoterpene synthase 7
ATGGCTCTTCATCAAATTTATTTCACTTCTTTCCCAGCAAGTTCCGTTGTCGAACGAGTCTATTCTTTTAAT
CACAACTCCTTATTTGTCCCTCGTGTGATGAATAATGTAACCAGTGGTGCAAGAATGTGTAGCCAGACTA
TTGTACGTCGATCGGGAAATTACCAACCTCCTACTTGGAAGCACGAGTTCATTCAATCATTAAGCAGTGA
ATTTGCGGGAGAAATATATGTAGGACGGTTTAATGAGTTAAAGGGAGAGGTAAGAGTCGTCATGAACA
AAATAGCTAACGACGATCCTCTAAAGCAACTCGAGTTTATTGATATCTTACAACGGCTTGGCATCTCATA
CCATTTCGAGAATGAGATTAAAGATATGTTGACAACAACATACAAGAAGTGTTGTGAAAATGACGATTG
GAAATGCAATAATCTCTATGCAACATCTCTTGAATTTAGACTTCTAAGACAACATGGATTCAATATTCCAC
AAGATATTTTTAATCCCTTCTTTTTTAACGAGACGAAAAGTTTCAATGTACAATTGTACGAAGATTTAAAA
GGAATGTTATATTTGTACGAAGCTTCATTCTTAAGTATAGAAGGCGAGAACATTTTAGAGACAGCAAAA
CATTTTACAAGGGAATATTTAGAAAAATACATGAAATCAAGCAAGGATGAAAATGAAGTAGCCATCGTT
AGACATGCATTAGAGCTTCCCTTGCATTGGAGAATGCCAAGATTGGAGACAAGATGGTTCATTGATATC

TATGAGAGAAAAGTGGATATGAATCCTATTCTTCTTGAATTTGCTAAACTTGATTTCAATAAGGTGCAAT
CTATCCACCAACAAGATTTGAAATATGCATCAAGGTGGTGGAGAAGAAGTGGACTTGGGGAAAAGTTG
AGCTTTGCAAGAGATAGATTGATGGAGAATTTCTTTTGGACAGTGGGTGTTGGATTTGAACCTGAACTTT
CTTATTTTAGAAGAATGACAACAAAAGTTAATGCACTAATAACAACTATAGATGATGTCTACGATGTTTA
TGGCTCATTAGATGAGCTCGAACTCTTTACCAGCGCAGTAGAGAGGTGGGATGTTGCTGCAATGGAAGA
GCTTCCTGACTATATGAAAATATGTTTTCTTGCTCTTCACAACTCTATAAATGAGATGGGTTTTGAAGCAC
TAAGAGACAAAGGAATTAATGTCATTCAATACCTTAAGAAAGCATGGATAGATTTGTGCAAAAGTTATA
TGTTGGAGGCAAAGTGGTATCACACCAATTATAAACCAACATTGGAAGAATATTTAGATAATGCATGGA
TTTCAATTTCAGGACCAGCTATATTAGTTCATGCATATGTTTTTGTCACATCTCCAACACTTGAAAATATG
GAGAGTTTGAAACAATATGTTGATATGATTCGTTGGTCATCGACAATTTTACGACTTGCTGATGATCTAG
GAACATCATCGGATGAATTAGAAAGAGGTGATGTTCCAAAATCAATACAGTGTTACATGAACGACACGG
GAGCTTCAGAGAATATTGCACGTGAATATATAGGGCATTTGATTGATGAGACGTGGAAAAAGTTAAATA
AGACTGAATTCGAGAATAATTATTCTATTTATCCTCGAGTGTTCATTGAGAGAGCTAAAAATCTTGCTAG
AATGGCTCAATGCATGTATCAATATGGCGATGGACATGGCATGGGATACCAAGAAACAAAAGATCGTGT
TATGTCTCTACTTATTGAACCGATTTCCATTCATTGTCACCATAGTGAATAA
>MTPS8 [organism=Cucumis melo] Melo3C023276 monoterpene synthase 8
ATGGCTCTTCATCAATTTCCCACTTCTAGTCAGACAAGTCGTTTCTTTGAACGAGTCTCTTGTAATTTCAAT
CCTTCATTTATGCCTCGAATCGTTAAAGCAATCAGTGTTACGACAAGAATAATGTGTAGTCGGACCATTG
TACGACGGTCGGGAAATTATCAACCTCCTATTTGGAAACATGAGTTCATTCAATCTTTGAGGAGCGAATT
TGCGGAAGAGATATATGTAGGACGCTTCAATGAGTTAAAAAGAGAAATACGACTCATCGTTAACCAAAT
AATTGATGATCCACTAAGGCAACTTGAGCTCGTTGATACATTACAACGACTTGGCATCTCATATCATTTTG
AGAACGAGATTAAGAATGTGTTGAAAACAGCGTTCGAGAAGAGCTATGAAAACGATTATTGGAAAAAG
AAGAATCTCTATGCTACATCACTTGAATTTCGACTTCTAAGACAACATGGATTCAATCTTTCACAAGATGT
TTTCAATAACTTCTACTCGGATGAGACAAAAAGTTTTAGCACACAATTGTACGAGGAGTTAAATGGAATA
TTATGTTTGCACGAAGCCTCATTCTTAAGTATAGAAGGCGAGAATATTTTAGAGACGGCAAAACATTTTA
CAATGGAATATTTAGAAAAATACATAAAATCAAGCAAGGATGAAAATGAAGTAGCCATTGTTAGACATG
CATTGGAGCTTCCCTTACATTGGAGAATGCCAAGATTGGAGACAAGATGGTTCATTGATATCTATGAGA
GAAAAGTGGATATGAATCCTATTCTTCTTGAATTTGCTAAACTTGATTTCAATAAGGTGCAATCGATCCAC
CAACAAGATTTGAAATATGCATCAAGTTGGTGGAGAAGCACCGGATTTGGAGAAAAGTTGAGCTTTGCA
AGAGATAGAGTGATGGAAAATTTCTTATGGACAGTAGGTTTTGGATATGAACCTGAATTCTCATTTTATA
GAAGAATGGCCACAAAAATTAATGCATTCATAACAACAATTGATGATGTTTATGATGTCTATGGCACATT
AGATGAACTCCAACTCTTTACCGACGCAATTGAGAGGTGGGATGTTGATGCATTGGACCAGCTTCCTGA
CTATATGAAAATATGTTTTCTTGCTCTCCACAATTCTATAAATGAGATGGCCTTTGAAGTATTAAGAGACA
AAGGAATTAATGTCATCCAATACCTTAAGAAAGCATGGGTAGATTTGTGCAAAAGTTACATGTTGGAGG
CAAAATGGTACCACACTAATTATAAACCAACATTGGAAGAATATTTAGATAATGCATGGATATCAATATC
AGGACCAGTTGTATTAGTTCATGCATATGTTTTTGTTACAACTCCAACACTTGAAAATATGGAGAGTTTG
AAACAATATGTTGATGTGATTCGTTGGTCATCAACAATTTTACGACTTGCAGATGATCTCGGAACTTCGT
CGGATGAATTAGCAAGAGGTGATGTTCCAAAATCAATACAATGTTACATGAACGATACAGGAAGCTCAG
AGAGTGACGCTCGTAAACATATAAGGCATTTGATTGATGAGACATGGAAGAAACTGAATAAGGTTCAA
GTCCAAAATTCTATCTTTCCTCAAGTGTTCATCGAGAGAGCTAAGAATGTTGCCAGAACGGCTCAATTCA
TGTATCAATATGGTGATGGACATGGCATAGGACACCAAGAAACAAAAGATCGTGTAATGTCTCTACTTA
TTCAACAGATTTCTATTCATCCCTATTGTGAAAAGTTGCTAAAGGTTATTTAG
>MTPS9 [organism=Cucumis melo] Melo3C023257 monoterpene synthase 9

ATGGCTCTTCACCAACTTTTCGCTACTTTTTCGATAAGTCCTTTCATCCAACGAATCTCTTTTAATTTTGATC
ATTTATGTGTGCATGTGCCTCGCACCTTTAAAGTTATTGGTGCTGCAACGATGTGTAATAAGACCATTGT
ACGACGATCGGGAAATTACCGACCTCCTATCTGGAAACACGAGTTTATCCAATCATTGAGAAGTGAATTT
GAGGAAGAAATATATATTGGACGATTCAATGAATTGAAGGGAGAAATAAGAGTCATCATGAACACAAT
AATTGACGATCCTTTAAAACAACTCGAGCTCATTGATATGTTACAACGACTTGGCATTTCATACCATTTTG
AGAATGAGATTAAGAACGTATTGAAAACAACATACGATACAAGCTATGAAAAGGAGCATTGGAAAAAC
AATAATCTCTATGCAACATCACTTGAATTTAGACTTCTAAGGCAACATGGATTCAATCTTTCACAAGATGT
TTTCAATCACTTCTTTTCGGATGAGACGAAAAGTTTCAACGTACAATTGTACGAGGATTTAAATGGAGTG
CTATATTTGTACGAAGCCTCATTTTTAAGTACAGAAGACGAGTACATTTTAGAGACAGCAAAACATTTTA
CAATGGAATATTTAGAAAAATACATGAAATCAAGCAAGGATGAAAATGAAGTAGCCATTGTTAGACATG
CATTGGAGCTTCCCTTGCATTGGAGAATGCCAAGATTGGAGACGAGATGGTTCATTGATATCTATGAGA
GAAAAGTGGATATGAATCCTATTCTTCTTGAATTTGCTAAACTTGATTTCAATAGGGTGCAATCTATCCAC
CAACAAGATTTGAAATATACATCAAGTTGGTGGAAAAGCAGCGGACTTGGGGAAAAATTGAGCTTTGCA
AGAAATAGGTTGATGGAGAATTTCTTATGGTCGGTAGGTTTTGGATATGAACCTGAATTCTCATATTACA
GAAGAATGGCTACAAAAATCAATGTATTTATAACAACAATTGATGATGTATATGATGTCTATGGCACATT
AGATGAACTCCAACTTTTTACCGATGCAATCGAGAGGTGGGATGTTGCTGCAATGGATGAGCTTCCTGA
CTACATGAAAATATGTTTTCTTGCTCTTCACAATTCTATAAATGAGATGGCTTTTGATGTACTAAGAGACC
AAGGAATTAATGTCATCCAATACCTTAAGAAAGTGTGGGTAGATTTGTGCAAAACTTATATGGTGGAGG
CAACATGGTACCACGATGGCTATAAACCAACATTGAAAGAATATCTAGATAATGCATGGACTTCAATATC
AGGACCGGTTATATTAGTTCATGCATATGTTTTCGTCACATCTCCAACCTTGAAGGATATGGAAAACTTG
AAACAGTACTTTGATTTGATTCGTTATTCGTCGACGATTTTACGACTTGCTGATGATCTAGGAACGTCATC
AGATGAATTAAAAAGAGGTGATGTTCCAAAATCAATACAATGTTATATGAACGATACAGGAGCTTCAGA
GAGTAATGCTCGAAAATATATAAAGCATTTGATTGATGAGACGTGGAAGAAGATGAATAAGATTGAAG
TAGAGAATCCTATTATCCCTCGAGTGTTTGTGGACAGAGCAAAGAATCTTGCTAGAATGGCACAATGTAT
GTATCAATATGGGGATGGACATGGCACAGCACATGAAGAAACAAAAGATCGTGTGATGTCTCTTCTTAT
TCAACCAATTTCTGTTCATTCATATGGTGAATAA
